# Investigation of Rapid Low-Power Microwave-Induction Heating Scheme on the Cross-Linking Process of the Poly(4-vinylphenol) for the Gate Insulator of Pentacene-Based Thin-Film Transistors

**DOI:** 10.3390/ma10070742

**Published:** 2017-07-03

**Authors:** Ching-Lin Fan, Ming-Chi Shang, Shea-Jue Wang, Mao-Yuan Hsia, Win-Der Lee, Bohr-Ran Huang

**Affiliations:** 1 Graduate Institute of Electro-Optical Engineering, National Taiwan University of Science and Technology, 43 Sec. 4, Keelung Road, Taipei 106, Taiwan; d10019004@mail.ntust.edu.tw (M.-C.S.); m10119016@mail.ntust.edu.tw (M.-Y.H.); huangbr@mail.ntust.edu.tw (B.-R.H.); 2 Department of Electronic Engineering, National Taiwan University of Science and Technology, 43 Sec. 4, Keelung Road, Taipei 106, Taiwan; 3 Institute of Materials Science and Engineering, National Taipei University of Technology, Taipei 106, Taiwan; sjwang@ntut.edu.tw; 4 Department of Electrical Engineering, Lee-Ming Institute of Technology, New Taipei 243, Taiwan; leewd@mail.lit.edu.tw

**Keywords:** organic thin-film transistor, pentacene, PVP, microwave, cross-linking, Microwave-Induction Heating (MIH)

## Abstract

In this study, a proposed Microwave-Induction Heating (MIH) scheme has been systematically studied to acquire suitable MIH parameters including chamber pressure, microwave power and heating time. The proposed MIH means that the thin indium tin oxide (ITO) metal below the Poly(4-vinylphenol) (PVP) film is heated rapidly by microwave irradiation and the heated ITO metal gate can heat the PVP gate insulator, resulting in PVP cross-linking. It is found that the attenuation of the microwave energy decreases with the decreasing chamber pressure. The optimal conditions are a power of 50 W, a heating time of 5 min, and a chamber pressure of 20 mTorr. When suitable MIH parameters were used, the effect of PVP cross-linking and the device performance were similar to those obtained using traditional oven heating, even though the cross-linking time was significantly decreased from 1 h to 5 min. Besides the gate leakage current, the interface trap state density (Nit) was also calculated to describe the interface status between the gate insulator and the active layer. The lowest interface trap state density can be found in the device with the PVP gate insulator cross-linked by using the optimal MIH condition. Therefore, it is believed that the MIH scheme is a good candidate to cross-link the PVP gate insulator for organic thin-film transistor applications as a result of its features of rapid heating (5 min) and low-power microwave-irradiation (50 W).

## 1. Introduction

In recent years, increased attention has been paid to organic thin-film transistors (OTFTs) because of their applicability in flexible displays, large-area chemical sensors for artificial skin applications, and radio-frequency power transmission devices [1,2,3]. To achieve better performance and more rapid carrier transport properties, gate dielectrics have become a prominent factor in improving gate leakage current, interfacial matching, and the orientation of semiconductor layers evaporated on dielectrics. Polymer gate dielectrics are the most promising candidates for use in OTFTs because of their solution process ability and low process temperature [4,5,6,7,8,9,10,11]. Among the family of polymer gate dielectrics, poly(4-vinylphenol) (PVP) has been reported most frequently because it allows high device performance to be achieved [4,5,6]. PVP is a polymer material with large molecular weight, which is difficult to process in an evaporator. Klauk et al. [12] reported that pentacene TFTs with spin-coated PVP gate dielectric layers had better electrical properties than TFTs with thermally grown S_i_O_2_ gate dielectric layers. However, the hydroxyl (–OH) groups in the PVP layer are known to be preferentially bonded with those in the linking agent through the curing step, which is called the cross-linking process. Therefore, an appropriate cross-linking technique must be carried out to reduce the –OH groups inside PVP dielectric films. In general, the cross-linking process usually uses an oven to heat the PVP film in order to reduce the –OH groups. However, the oven heating process usually requires a curing temperature of 100 °C–300 °C [4,5,6,7,8] and needs a longer treatment time (1–2 h). Recently, several research groups have published alternative routes that lower the cross-linking temperature by adding UV-light [13], by incorporating silanes in the solution [14], or by using a different cross-linker [15,16] or PVP derivatives [17]. However, an efficient curing time is still a significant issue that should be investigated.

Traditional microwave annealing is known to be effective for heating because of its advantages including a rapid heating process, shortened manufacturing period, damage-free process and low thermal budget [18,19]. In the reported papers, microwave annealing schemes were always used for heating the semiconductor film because some materials cannot absorb the microwave energy, such as insulators. Therefore, materials heated by using microwave radiation will be limited by the selective-heating characteristic of microwaves. In addition, the high microwave power was also a serious issue, leading to a trade-off between fabrication cost and device performance [18,19,20,21]. In a previous study [22], a Microwave-Induction Heating (MIH) scheme was proposed for the PVP gate insulator cross-linking process to replace the traditional oven heating process. However, systematic studies must be carried out to acquire suitable MIH parameters. Therefore, the detailed MIH scheme and suitable parameters were studied in this work. It was found that these MIH parameters including chamber, microwave power, and heating time should be optimized and traded-off to achieve the optimal device performance and fabrication cost. The suitable MIH parameters were found to be a power of 50 W, heating time of 5 min, and a pressure of 20 mTorr. The effect of PVP cross-linking using the proposed MIH scheme was similar to that using traditional oven heating, even though the cross-linking time was significantly decreased from 1 h to 5 min. Therefore, the MIH scheme is a good candidate to replace traditional thermal heating for the PVP gate insulator cross-linking process.

## 2. Device Fabrication

A glass substrate with an indium tin oxide (ITO) layer was used as a substrate and as a bottom gate electrode. PVP powder was mixed with poly(melamine-co-formaldehyde) methylated (PMF), working as a cross-linking agent, in propylene glycol monomethyl ether acetate (PGMEA). The mixed solution was then spin-coated onto the substrate to form a 600-nm-thick insulating layer. MIH was subsequently used for heating in the cross-linking step. The PVP was also cross-linked in a vacuum oven at 180 °C for 1 h as the control sample. A 50-nm-thick pentacene layer was then deposited as a channel layer onto the PVP layer using a thermal evaporator with the substrate temperature maintained at room temperature. Gold source/drain contacts were deposited through a shadow mask onto the pentacene channel layer using a thermal evaporator. The MIH power was varied from 50 to 150 W, the chamber pressure from 2000 to 20 mTorr, and the heating time was also varied from 20 min to 2.5 min. The gate leakage current was measured and the interface trap state density (Nit) was also calculated to describe the interface status between the gate insulator and the active layer. All devices were measured in the final step using a semiconductor parameter analyzer (HP 4145B, Hewlett-Packard, Palo Alto, CA, USA). In addition, the Fourier transform infrared (FTIR, Shimadzu, Tokyo, Japan) and the atomic force microscope (AFM, Bruker, Billerica, MA, USA) were used to measure the amount of –OH groups inside the PVP and the surface morphology of PVP, respectively. The field-effect mobility (*μ*_FE_) of the pentacene-based OTFTs was estimated from the saturation region using Equation

(1) $$I_{D}=\frac{W\mu_{\mathrm{FE}}c_{ox}}{2L}{\left(V_{\mathrm{GS}}-V_{\mathrm{th}}\right)}^{2}$$

where *μ*_FE_ is the field-effect mobility, *C_ox_* is the capacitance density of the gate insulator, *V*_th_ is the threshold voltage, and *W* (width) and *L* (length) are the dimensions of the semiconductor channel defined by the source and drain electrodes. The maximum and minimum values of drain current (*I*_D_) at a drain voltage (*V*_DS_) of −20 V are designated as *I*_on_ (on-current) and *I*_off_ (off-current), respectively. 

## 3. Results and Discussion

**Figure 1:**
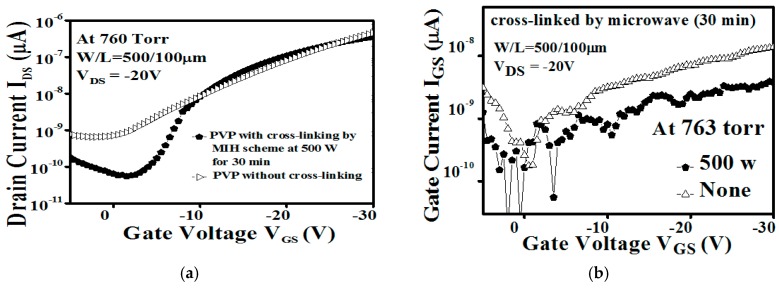
(**a**) The transfer characteristics (*V*_DS_ = −20 V); (**b**) The gate leakage current of the organic thin-film transistor (OTFT) with the poly(4-vinylphenol) (PVP) gate insulator cross-linked using the Microwave-Induction Heating (MIH) scheme at 500 W for 30 min under a pressure of 763 Torr and the OTFT with the pure PVP gate insulator (without cross-linking), respectively. (W/L = 500 µm/100 µm).

Figure 1 illustrates the *I*_DS_–*V*_GS_ of the OTFT with a PVP gate insulator cross-linked using the MIH scheme at 500 W for 30 min under a pressure of 760 Torr and the *I*_DS_–*V*_GS_ of the OTFT with a pure PVP gate insulator without any cross-linking. The related gate leakage currents are shown in Figure 1b. The device with the PVP cross-linked by MIH presents obvious transfer characteristics. On the contrary, poor electrical characteristics with a high gate leakage current can be found in the OTFT with pure PVP without cross-linking. Thus, the proposed MIH scheme can be used successfully for the heating process of the PVP insulator.

**Figure 2:**
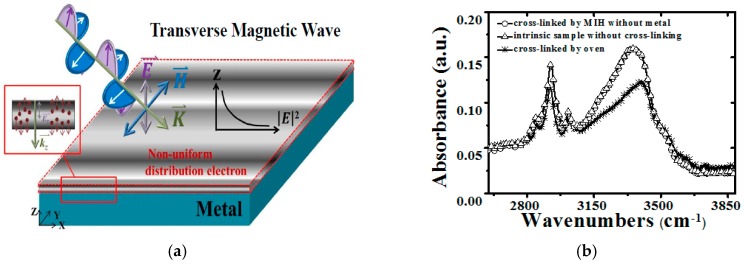
(**a**) Schematic diagram of the microwave-induction heating model; (**b**) The Fourier transform infrared (FTIR) spectra of PVP films with the MIH scheme or oven heating cross-linking and without cross-linking, respectively.

Figure 2a illustrates a schematic diagram of the proposed MIH scheme for the PVP cross-linking process. When microwaves were incident on the conductor, the electric field decayed rapidly at the conductor surface because of the skin effect. The electric field at the conductor surface can induce a non-uniform charge distribution inside the conductor, causing a surface current. The surface current crowded at the surface will immediately induce a high temperature because the conductor acts like a thin resistor due to the ultra-thin skin depth. Therefore, when the PVP with the ITO below is irradiated by microwaves, the waves pass through the PVP gate insulator to the ITO metal surface. The thin ITO metal is heated rapidly by the microwave irradiation. The heated ITO metal gate will heat the PVP gate insulator, resulting in the cross-linking of the PVP (called microwave induction heating, MIH). In this study, the ITO was selected as the gate metal because of its transparency. If the MIH scheme can only be conducted for 5 min for the used ITO, we presume that the time of MIH will be less than 5 min as a result of the low metal conductivity when we use other metals. Figure 2b shows the FTIR measurement of the PVP insulator with cross-linking by microwave irradiation or oven heating without the ITO metal below the PVP insulator. The PVP insulator fabricated without any cross-linking step is also measured by FTIR as the intrinsic sample. This shows that the PVP insulator can be cross-linked using traditional oven heating. However, it was also found that the –OH group peak for the PVP insulator without ITO metal below is almost the same as that for the intrinsic sample, which indicates that the cross-linking process cannot be carried out by MIH if there is no ITO metal beneath the PVP insulator. In addition, it is believed that the power of 500 W is too high and the irradiation time of 30 min is too long and therefore they cannot meet the requirements regarding of the manufacturing and energy savings. Thus, it is necessary to discuss the trade-off of these parameters, namely the chamber pressure, microwave power, and heating time.

**Figure 3:**
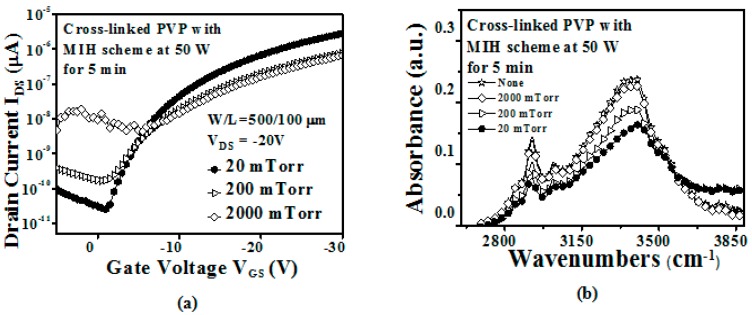
(**a**) The transfer characteristics (*V*_DS_ = −20 V) of the OTFT with the PVP gate insulator cross-linked using different values of MIH power (50 W) under different pressures (2000, 200, and 20 mTorr) for 5 min (W/L = 500 µm /100 µm); (**b**) The FTIR measurement of the sample with the PVP gate insulator cross-linked using the MIH of 50 W under different pressures (2000, 200, and 20 mTorr) for 5 min.

Figure 3a shows the transfer curves of devices with the PVP gate insulator cross-linked using an MIH power of 50 W under different pressures (2000, 200, and 20 mTorr) for 5 min. It was found that the device performance was degraded with increased microwave chamber pressure, as a result of the increased microwave attenuation. We presume that the MIH efficiency decreases significantly due to the increased chamber pressure, causing an inferior cross-linking effect on the PVP gate insulator, resulting in inferior device characteristics. Figure 3b shows the FTIR measurement of the sample with the PVP gate insulator cross-linked by MIH (50 W) under different pressures (2000, 200 and 20 mTorr) for 5 min. It was found that the –OH group peak for the PVP gate insulator cross-linked under 2000 mTorr is almost the same as that for the intrinsic sample. This means that the cross-linking efficiency is significantly inferior and it can be noted that MIH does not occur when MIH power of 50 W is used under 2000 mTorr. In addition, it is found that the –OH group peak decreases with decreasing microwave chamber pressure. It is confirmed that the MIH efficiency obviously increases when the microwave chamber pressure deceases. We presume that the MIH efficiency was significantly increased under 20 mTorr as a result of the reduction in the density of the gas molecules. The increasing chamber pressure results in a high density of gas molecules, causing attenuation of the microwave energy as a result of the absorption of the microwave energy by the gas molecules. Thus, it is believed that reduction of the chamber pressure is necessary to decrease the attenuation of the microwave energy and thus to achieve a power of 50 W and a heating time of 5 min for the MIH scheme.

**Figure 4:**
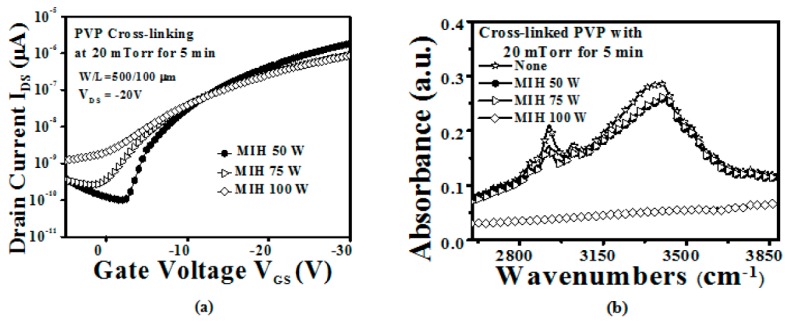
(**a**) The transfer characteristics (*V*_DS_ = −20 V) of the OTFT with the PVP gate insulator cross-linked using different MIH powers (50, 75, 100, and 150 W) under 20 mTorr for 5 min (W/L = 500 µm/100 µm); (**b**) The FTIR measurement of the sample with the PVP gate insulator cross-linked using different MIH powers (50, 75, 100, and 150 W) under 20 mTorr for 5 min.

**Figure 5:**
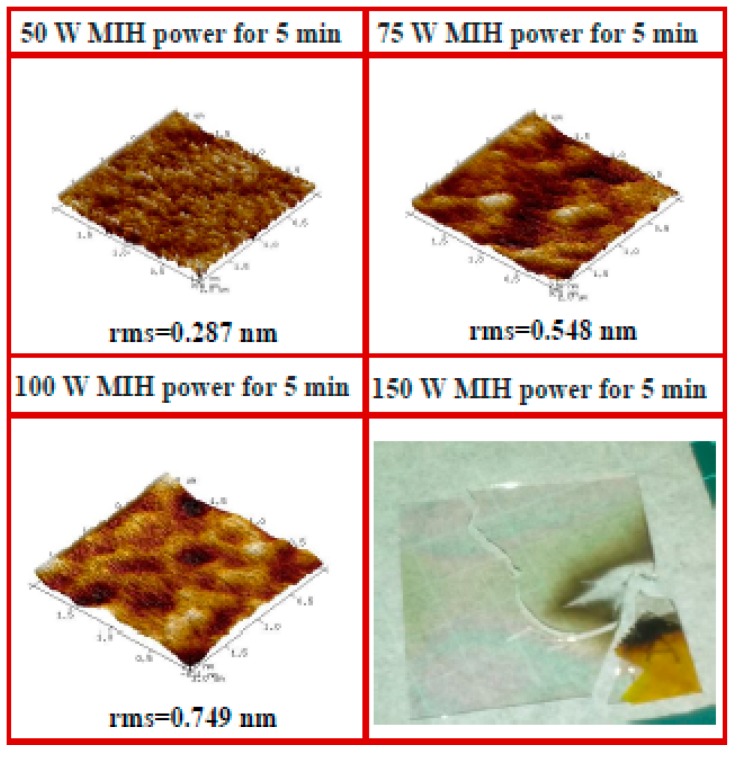
The atomic force microscope (AFM) surface image of the PVP gate insulator cross-linked with different MIH powers (50, 75, 100, and 150 W) under 20 mTorr for 5 min.

Figure 4a shows the transfer curves of devices with the PVP gate insulator cross-linked using different values of MIH power (50, 75, and 100 W) under 20 mTorr for 5 min. A significant degradation of the device characteristics was found when the MIH power was increased. Figure 4b illustrates the FTIR measurement of the sample with the PVP gate insulator cross-linked using different values of MIH power (50, 75, and 100 W) under 20 mTorr for 5 min. The MIH power of 100 W is too high and will damage the PVP film. Therefore, the –OH group peak cannot be observed when the MIH power is equal to 100 W under 20 mTorr, as shown in Figure 4b. The atomic force microscope (AFM) surface image was used to measure the surface morphology of the cross-linked PVP gate insulator, as shown in Figure 5. It shows that the surface roughness increased as the MIH power increased. It can also be observed that the PVP surface seems to melt when the MIH power exceeds 75 W. The melt-like PVP surface will result in clearly increased roughness. This means that the thermal energy conducted by MIH not only conducts the cross-linking process but also causes the change in the PVP surface morphology. Therefore, we presume that the increased PVP surface roughness and the changed surface morphology cause degradation of the device characteristics, as shown in Figure 4a. In addition, the sample with MIH of 150 W under 20 mTorr will be seriously damaged and broken as a result of overheating the PVP film. It can be noted that the low microwave power of 50 W is preferred to the MIH scheme under a pressure 20 mTorr and a heating time of 5 min.

**Figure 6:**
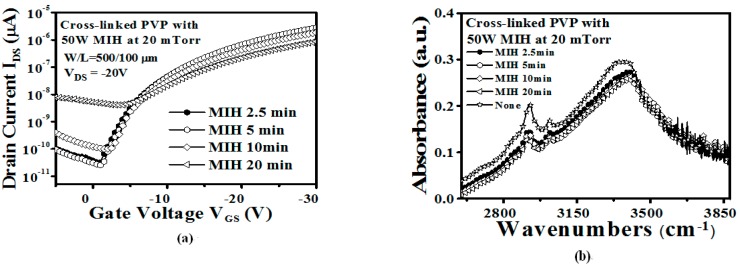
(**a**) The transfer characteristics (*V*_DS_ = −20 V) of the OTFT with the PVP gate insulator cross-linked using MIH power of 50 W under a pressure of 20 mTorr for different cross-linking times (2.5, 5, 10, and 20 min) (W/L = 500 µm /100 µm); (**b**) The FTIR measurement of the sample with the PVP gate insulator cross-linked using MIH power of 50 W under a pressure of 20 mTorr for different cross-linking times (2.5, 5, 10, and 20 min).

**Figure 7:**
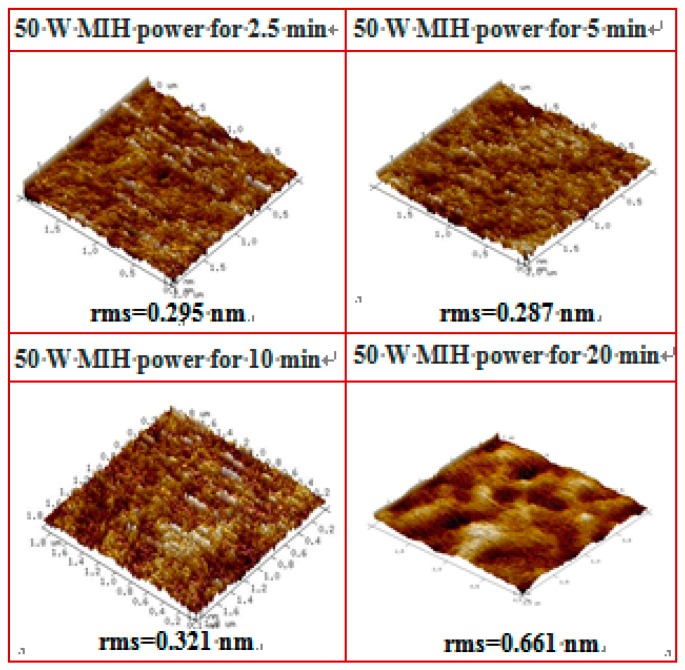
The atomic force microscope (AFM) surface image of the PVP gate insulator cross-linked with MIH power of 50 W under a pressure of 20 mTorr for different cross-linking times (2.5, 5, 10, and 20 min).

Figure 6a shows the transfer curves of devices with the PVP gate insulator cross-linked using MIH power (50 W) under 20 mTorr for different cross-linking times (2.5, 5, 10, and 20 min). The devices’ characteristics obtained with the PVP insulator cross-linked for 5 min were superior to those obtained with increased MIH times. The FTIR measurements of samples with the PVP gate insulator cross-linked using MIH power of 50 W under 20 mTorr for different cross-linking times (2.5, 5, 10, and 20 min) are shown in Figure 6b. When the cross-linking times are longer than 5 min, the decrease of the –OH group peak presents a saturation situation, as shown in the inset of Figure 6b. In addition, with increases in MIH time, the devices present a high leakage current. Figure 7 depicts the AFM surface image of the cross-linked PVP gate insulator with different cross-linking time (2.5, 5, 10, and 20 min) at 50 W under 20 mTorr. The longer cross-linking time will result in high thermal energy, causing a melt-like PVP surface (20 min), which results in increased surface roughness. The increased surface roughness between the insulator and the channel layer will increase the leakage current and degrade the device performance, as shown in Figure 6a. It shows that the MIH for 5 min under a pressure of 20 mTorr at a power of 50 W provides the suitable conditions for the PVP cross-linking process without performance degradation in the study.

**Figure 8:**
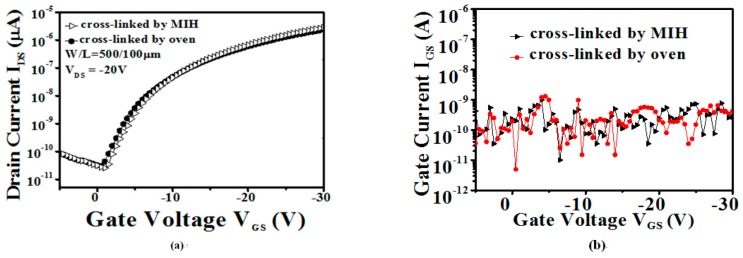
(**a**) The transfer characteristic (V_DS_ = −20 V); (**b**) The gate leakage current (I_GS_) of the OTFT with the PVP gate insulator cross-linked using the proposed MIH at 50 W for 5 min under a chamber pressure of 20 mTorr and using a vacuum oven at 180 °C for 1 h, respectively (W/L = 500 µm/100 µm).

Figure 8a compares the device characteristics of the OTFTs with the PVP gate insulator cross-linked using suitable parameters of the MIH scheme (50 W, 5 min, 20 mTorr) to those obtained using vacuum oven heating at 180 °C for 1 h, respectively. The device characteristics obtained with the PVP cross-linked using MIH are comparable to those of the oven-heated sample. That is to say, the effect of PVP cross-linking by using the proposed MIH scheme was similar to that using traditional oven heating, even though the cross-linking time was significantly decreased from 1 h to 5 min. The *μ*_sat_ of the devices with MIH cross-linking of the PVP gate insulator was 0.44 cm^2^/V·s. Moreover, *V*_th_ of −8.5 V and an acceptable *I*_on_/*I*_off_ of 1.1 × 10^5^ were also acquired, as listed in Table 1. Figure 8b illustrates the gate leakage current (*I*_G_) of the OTFT device with the PVP gate insulator cross-linked using the proposed MIH under the optimal conditions and using oven heating at 180 °C for 1 h, respectively. Similar gate leakage currents can be observed for both samples. The gate leakage current depends on the vertical electric field (*V*_GS_) and the quality of the PVP gate insulator. Thus, it should be a factor for evaluating the quality of the gate insulator. It should be noted that the MIH cross-linking process did not degrade the PVP insulator performance, as compared to the oven heating process. Besides the gate leakage current, the interface trap state density (Nit) is another factor that can describe the interface status between the gate insulator and the active layer and it can be calculated using the following equation (where *S.S.*, *k*, *T*, and *C*_ox_ represent the sub-threshold swing, Boltzmann’s constant, absolute temperature, and insulator capacitance per unit area, respectively).

(2) $$N_{it}=\left[\frac{S.S.\cdot \log(e)}{kT/q}-1\right]\cdot \frac{C_{ox}}{q}$$

In the above equation, the sub-threshold swing (*S.S.*) is a measure of how rapidly the device switches from the off state to the on state, and is typically reported in V/decade or mV/decade. Furthermore, the *S.S.* also represents the interface quality and the trap density. The *S.S.* can be extracted from the inverse sub-threshold slope versus the *V*_GS_ curve using the following equation:

(1) $$S.S.={\left[\frac{\partial \left(\log I_{DS}\right)}{\partial V_{GS}}\right]}^{-1}$$

It is found that the interface trap state densities are 8.86 × 10^11^ cm^−2^, 2.42 × 10^12^ cm^−2^, and 4.48 × 10^12^ cm^−2^ for 50 W, 75 W 100 W, respectively. The interface trap state density is 9.18 × 10^11^ cm^−2^ for the device treated by oven heating. The lowest interface trap state density can be found in the device with the PVP gate insulator cross-linked by using the proposed MIH at 50 W for 5 min. It is also shown that the interface trap state density increases with the increasing used power, which is a similar trend to the increased surface roughness. We presume that the increased surface roughness will increase the interface trap density as a result of the increased MIH power. From the above discussion, these parameters, namely the chamber pressure, microwave power, and heating time were optimized to achieve a device performance comparable to that achieved with traditional oven-heating even though the heating time was significantly decreased from 1 h to 5 min. In summary, the proposed MIH scheme can be carried out accurately under optimal conditions for the PVP gate insulator cross-linking process in OTFT applications. The important electrical parameters in this study are also listed in Table 1. It is believed that the MIH scheme is a good candidate for the PVP gate insulator cross-linking process due to its rapid heating (5 min) and low power microwave-irradiation (50 W) features.

## 4. Conclusions

The proposed Microwave-Induction Heating (MIH) scheme has been systematically studied and suitable MIH parameters have also been determined. The proposed MIH scheme means that the thin ITO metal below the PVP film is heated rapidly by microwave irradiation and the heated ITO metal gate can heat the PVP gate insulator, resulting in PVP cross-linking, even if the microwave energy cannot be absorbed by the PVP gate insulator. It was also found that the decreased chamber pressure was necessary to decrease the attenuation of microwave energy. In this study, a chamber pressure of 20 mTorr, the power of 50 W and heating time of 5 min were the suitable conditions for the cross-linking process of the PVP gate insulator. By using the suitable MIH parameters (power, pressure and time), the effect of PVP cross-linking using the proposed MIH scheme was similar to that achieved using traditional oven heating, even though the cross-linking time was significantly decreased from 1 h to 5 min. In addition, the device characteristics obtained with the suitable MIH conditions are comparable to those obtained with conventional oven heating. Therefore, the proposed MIH scheme can be carried out successfully for the PVP gate insulator cross-linking process for OTFT applications. We suggest that the MIH scheme is a good candidate to replace traditional thermal heating for cross-linking of PVP as the gate insulator for organic thin-film-transistors.

## Figures and Tables

**Table 1:** Important electrical parameters of the OTFT with the PVP gate insulator cross-linked using different MIH conditions in this study.

OTFT with PVP gate insulator cross-linked using different MIH power (50 W) with different pressures (2000, 200, 20 mTorr) for 5 min (W/L = 500 μm/100 μm).					
	***μ*_sat_**	***I*_on_/*I*_off_**	***S.S.***	***V*_th_**	**(Nit)**
**2000** **mTorr**	0.114	1.8 × 10^2^	3.85	−10.14	3.72 × 10^12^
**200** **mTorr**	0.118	4.45 × 10^3^	2.90	−8.877	1.95 × 10^12^
**20** **mTorr**	0.448	1.11 × 10^5^	1.35	−8.544	8.86 × 10^1^^1^
OTFT with PVP gate insulator cross-linked using different MIH power (50, 75, 100, 150 W) with 20 mTorr for 5 min (W/L = 500 μm/100 μm).					
	***μ*_sat_**	***I*_on_/*I*_off_**	***S.S.***	***V*_th_**	**(Nit)**
**50 W**	0.448	1.11 × 10^5^	1.35	−8.544	8.86 × 10^1^^1^
**75 W**	0.124	3.43 × 10^3^	3.58	−5.077	2.42 × 10^12^
**100 W**	0.123	7.17 × 10^2^	6.59	−5.773	4.48 × 10^12^
OTFT with PVP gate insulator cross-linked using MIH power (50 W) under 20 mTorr for different cross-linked time (2.5, 5, 10, 20 min) (W/L = 500 μm/100 μm).					
	***μ*_sat_**	***I*_on_/*I*_off_**	***S.S.***	***V_t_*_h_**	**(Nit)**
**2.5 min**	0.437	8.30 × 10^4^	1.55	−8.574	1.03 × 10^12^
**5 min**	0.448	1.11 × 10^5^	1.35	−8.544	8.86 × 10^1^^1^
**10 min**	0.315	1.85 × 10^4^	1.61	−10.36	1.07 × 10^12^
**20 min**	0.141	2.11 × 10^2^	6.96	−7.757	4.74 × 10^12^
OTFT with PVP gate insulator cross-linked using Oven (180 °C for 1 h) (W/L = 500 μm/100 μm)					
	***μ*_sat_**	***I*_on_/*I*_off_**	***S.S.***	***V*_th_**	**(Nit)**
**Oven**	0.425	7.9 × 10^4^	1.77	−8.04	9.18 × 10^11^
